# Diagnostic Difficulties in a Case of Fetal Ventricular Tachycardia Associated with Neonatal COVID Infection: Case Report

**DOI:** 10.3390/ijerph182312796

**Published:** 2021-12-04

**Authors:** Liliana Gozar, Carmen Corina Șuteu, Dorottya Gabor-Miklosi, Andreea Cerghit-Paler, Amalia Făgărășan

**Affiliations:** 1 Pediatric Cardiology Department, Emergency Institute for Cardiovascular Diseases and Transplantation, 540136 Târgu-Mureș, Romania; liliana.gozar@umfst.ro (L.G.); annadorka@yahoo.com (D.G.-M.); palerandreea@yahoo.com (A.C.-P.); amalia.fagarasan@umfst.ro (A.F.); 2 Department of Pediatrics, “George Emil Palade” University of Medicine, Pharmacy, Science, and Technology of Târgu-Mureș, 540139 Târgu-Mureș, Romania

**Keywords:** COVID, coronavirus, newborn, myocarditis, fetal tachycardia, arrhythmic storm, case report, pediatrics

## Abstract

The clinical course of COVID in the pediatric population is considered to be much milder when compared to adults; however, the occurrence of severe and fatal forms of the disease in children is non-negligible, especially in patients with comorbidities such as prematurity or cardiac disease. We report a case of a newborn with sotalol-controlled fetal ventricular tachycardia, who was postnatally diagnosed with COVID infection. The myocardial injury was sustained on the basis of pericardial effusion, left ventricular dysfunction, rapid progression to coronary artery dilation, and an arrhythmic storm. We believe that, in our case, there is a significant overlap between fetal ventricular tachycardia, associated with impaired left ventricular function, and COVID infection, diagnosed after birth; both factors contribute to the myocardial dysfunction with a fulminant clinical evolution. To our knowledge, this is the first case describing neonatal myocardial dysfunction associated with SARS-CoV infection complicating the clinical course of rare fetal tachyarrhythmia.

## 1. Introduction

The clinical course of COVID (caused by the severe acute respiratory syndrome coronavirus-2—SARS-CoV) in the pediatric population is much milder than in adults [1,2]. Data suggest that a large spectrum of clinical presentations, as well as the non-negligible occurrence of severe and fatal forms of COVID in children, mostly occur in patients with comorbidities [3,4]. The most common comorbidities associated with severe forms of the disease are prematurity, respiratory and cardiac comorbidities, and obesity [4]. SARS-CoV infection has been associated with myocardial dysfunction and heart failure in adult patients [5,6]. In the pediatric population, cardiac complications occur both in the acute phase of infection and, especially, in the post-acute phase, when a small number of children develop a severe condition called multisystem inflammatory syndrome in children (MIS-C) [4,7]. Grimaud M et al. observed that acute myocarditis associated with SARS-CoV infections are less severe than those usually observed- in children, and are characterized by intense systemic inflammation, Kawasaki-like symptoms, and vasoplegia [2].

A limited number of cases of myocarditis in SARS-CoV infection in newborns are reported in the literature [8]. We present the clinical course of myocardial dysfunction associated with SARS-CoV infection in a newborn diagnosed with fetal ventricular tachycardia (VT), a rare form of fetal tachyarrhythmia. Early diagnosis and transplacental therapy with sotalol are associated with a good postnatal prognosis in this malignant fetal arrhythmia [9]. As an antiarrhythmic agent, sotalol possesses both non-selective beta-adrenergic antagonist and potassium channel blocker properties, thus prolonging cardiac repolarization.

## 2. Case Description

**Figure 1:**
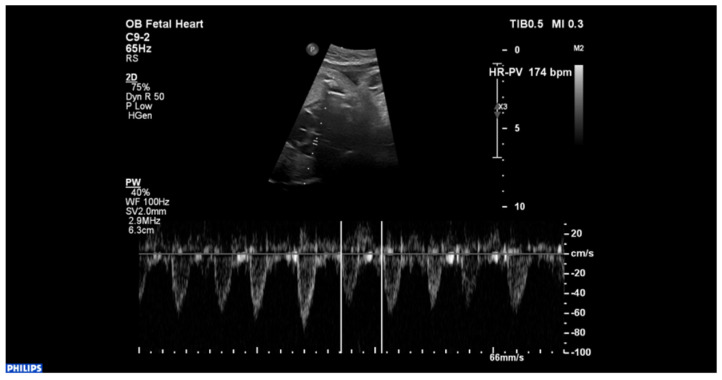
Fetal echocardiogram displaying couplets of premature ventricular beats.

The patient in our case report is a female infant, born from a 39-week-pregnant woman with a history of fetal dysrhythmia, diagnosed at 33 weeks of gestation. Upon echocardiographic evaluation, the M-mode and pulsed-wave Doppler showed couplets of premature ventricular beats (Figure 1).

**Figure 2:**
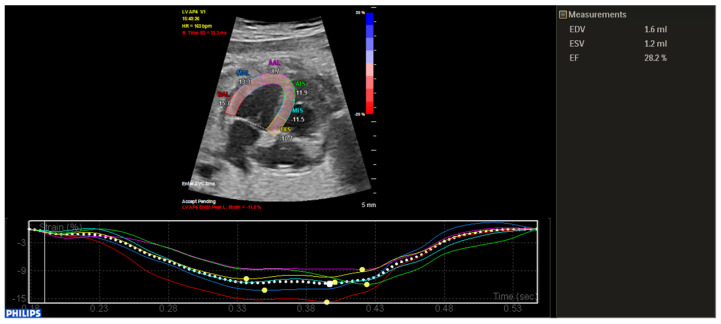
Fetal speckle tracking showing decreased ventricular function.

During evolution, due to a decrease in contractile function, quantified by left ventricular ejection fraction and global longitudinal strain (Figure 2), as well as the appearance of pericardial effusion, the decision to initiate antiarrhythmic medication with sotalol was made.

The intrauterine evolution was favorable later on, with the maintenance of sinus rhythm and normalization of ventricular function.

There was no history of symptoms, or exposure to a suspected SARS-CoV case in the mother’s family. An emergency caesarean section was performed because of the fetal distress, and a female infant with a weight of 3370 g and height of 50 cm was delivered. The newborn was hemodynamically stable, and, at that time, she did not require admission to the neonatal intensive care unit (NICU).

The 12-lead electrocardiogram (ECG) performed immediately after birth revealed sinus rhythm, normal QRS voltage, QTc: 380 ms, and the first echocardiography showed normal left ventricular (LV) ejection fraction, without left and right ventricular dilation. The origins of the coronary arteries were normal.

**Figure 3:**
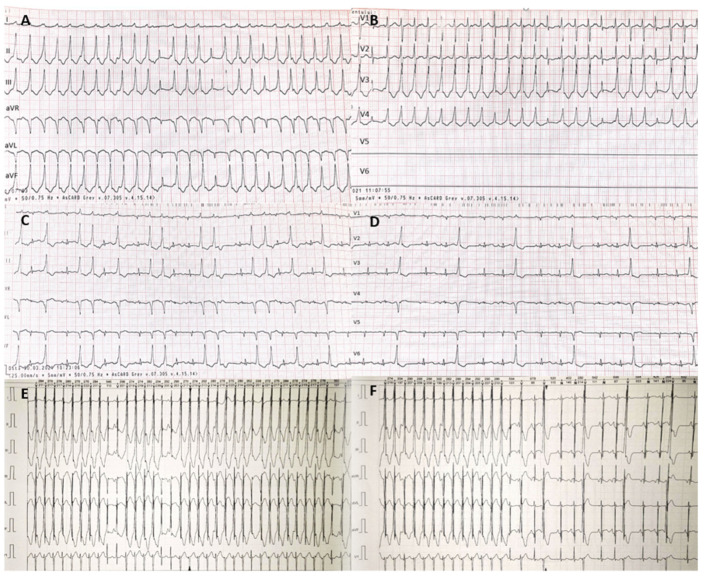
Electrocardiograms and 24-h electrocardiograms. (**A**,**B**)—electrocardiograms showing ventricular tachycardia-dissociated activity of atria and ventricles, ventricular rate: 220–230/min, fusion beats and capture beats; (**C**,**D**)—electrocardiograms showing ventricular bigeminy and trigeminy; (**E**,**F**)—24-h electrocardiograms showing ventricular tachycardia.

On the third day after birth, the ECG revealed sinus rhythm, but the 24-h ECG showed recurrent repetitive monomorphic VT with short periods of sinus rhythm. There was a slightly widened QRS (QRS: 85 ms) with a pattern resembling a partial left bundle branch block, and an inferior QRS axis. The ventricular rate was 220–230/min, the P waves were visible intermittently, with dissociated activity of the atria and ventricles, and with evidence of fusion and capture beats (Figure 3).

The newborn was hemodynamically stable; however, she was transferred to the NICU. Despite propranolol treatment, sinus rhythm was not obtained, thus amiodarone was initiated for pharmacological cardioversion, initially with partial control of the VT. The second echocardiography showed minimal pericardial effusion with no clear signs of hemodynamic deterioration.

On the fifth day after delivery, the diagnosis of SARS-CoV infection was confirmed for the mother and the neonate by real-time reverse transcription polymerase chain reaction (rRT-PCR) using a nasopharyngeal specimen.

Due to the clinical picture indicating myocardial injury (pericardial effusion, VT was highlighted on the third day after birth, after the transplacental treatment with sotalol had been proven successful, and documentation of COVID infection had been obtained), in addition to the usual laboratory investigations, the following were performed: chest X-rays, high-sensitivity troponin (TnI), N-terminal fraction of pro-brain natriuretic peptide (NT-proBNP), ferritin, D-dimer, and lactate measurements. The laboratory data showed a normal level of lymphocytes (3.23 × 10^3^/μL), elevated TnI 25.7 pg/mL (normal < 15.6 pg/mL), elevated D-dimer 729 ng/mL (normal < 400 ng/mL), and elevated C-reactive protein (CRP) 26.00 mg/L (normal < 0.5 mg/dL). Neither bacterial nor non-SARS-CoV viral infections were identified. Chest radiography showed a normal pattern of the lung tissue.

Table 1 shows the trend of selected laboratory values.

Between the fifth and thirteenth day after the confirmation of COVID infection, there was a progressive increase in the serum levels of TnI, D-dimer, and ferritin. The serial echocardiographies revealed persistence of the pericardial effusion, progressive dilation of the left coronary artery (left main coronary artery z score: 2.71, left anterior descending coronary artery z score: 3.9) with diffuse myocardial edema in the basal-anteroseptal, basal-inferoseptal, basal-inferior, mid-inferoseptal, and mid-inferior LV segments, with regional wall motion abnormalities, initially with preserved LV ejection fraction. On the twelfth day of COVID infection, the echocardiography showed LV systolic dysfunction (ejection fraction of 30%), correlated with the increased serum NT-proBNP level of 645.40 pg/mL. The 24-h ECG showed sinus tachycardia with ventricular bigeminy and trigeminy, and very frequent ventricular couplets. It was necessary to increase the dose of amiodarone to 15 μg/kg/min. The serial ECGs revealed the progression of the Q-wave amplitude in the inferior leads and nonspecific ST-T wave changes.

Based on the clinical course, elevated TnI, ECG changes, and echocardiographic changes (pericardial effusion, regional wall motion abnormalities with decreased LV function, and coronary anomalies), we considered a possible inflammatory myocardial injury associated with COVID infection. Myocarditis treatment was started with intravenous immunoglobulin (2 g per kilogram) on the fifth and sixth day of the illness. In the presence of coagulopathy, low-molecular-weight heparin was initiated at a therapeutic dose. Echocardiographic evidence of coronary dilation required antiplatelet therapy. Considering the inflammatory syndrome, intravenous dexamethasone at 0.5 mg/kg/day was started on the ninth day of COVID infection. In the presence of clinical signs of heart failure and echocardiographic evidence of systolic and diastolic LV dysfunction, diuretic therapy with furosemide and spironolactone was initiated. The inotropic support with levosimendan (loading dose of 0.1 μg/kg/min) was infused on the twelfth and thirteenth day after the confirmation of COVID infection.

The subsequent clinical evolution was favorable, with a substantial decrease in inflammatory biomarkers, and normalization of TnI, NT-pro-BNP, and D-dimer after 3 weeks of treatment. The repeated 24-h ECG showed sinus rhythm. The infant was discharged with LV systolic function recovery. At the time of this submission, the patient had recovered and was discharged home on oral heart failure therapy with a close follow-up.

The publication of this case was approved by the institutional review board in the presence of signed informed consent obtained from the mother.

## 3. Discussion

To date, a number of pediatric cases of SARS-CoV infection have been reported, including neonatal infection. Amiraskari R et al. pointed out the different transmission routes for neonatal infections, including the following: vertical transmission from the mother to the fetus during pregnancy, contact between maternal blood and body fluid during a caesarean, and contact transmission of newborns in the hospital [8]. Yang Pu. et al. concluded that the infection of SARS-CoV in late pregnancy does not cause adverse outcomes in newborns; however, it is necessary to separate newborns from mothers immediately to avoid the potential threats [10]. Due to our limited financial resources, SARS-CoV tests are not routinely performed on admission. Therefore, in our case, the transmission route of the infection to the newborn can not be specified, due to the fact that both the infant and mother were confirmed to be positive on the fifth day after birth and their separation took place two days before the confirmation of the infection.

The pathophysiology of this virus is still unknown. The effect of SARS-CoV infection on myocardial function is still not well established as there are no robust histological data. Cardiac complications are described in the acute phase of infection, but are more common in the post-acute phase [4]. A possible new spectrum of vasculitis and inflammatory diseases following SARS-CoV infection, rather than direct viral organ damage, may be involved in the pathophysiology of myocardial dysfunction [2]. Guo T. et al. concluded that myocardial injury is associated with cardiac dysfunction and arrhythmias, respectively, and inflammation may be a potential mechanism for myocardial injury [6]. The serum levels of IL-6, NT-proBNP, and TnT are higher in these patients [5]. Clinical manifestations of children’s COVID cases were generally less severe than those of adult patients [11,12]. Sharma M et al. reported a case of reversible myocardial injury and heart failure in an infant with SARS-CoV infection, in which the acute myocardial injury was defined by severe LV dysfunction and elevated cardiac biomarkers (TnT and NT-proBNP) [13]. Kesici S et al. reported fulminant SARS-CoV-related myocarditis in an infant, in which the biopsy specimen of the myocardium, taken during ECMO cannulation, showed the presence of the viral genome in the myocardial tissue together with local inflammation [14]. A limited number of newborns with myocardial injury associated with SARS-CoV have been reported [8,15,16].

We report the case of a newborn with sotalol-controlled fetal VT at 33 weeks of gestation, who was diagnosed with SARS-CoV infection on the fifth day after birth. Ventricular tachycardia is a rare cause of tachycardia during fetal life and occurs in less than 2% of fetal tachyarrhythmia cases [9]. The prognosis depends on the underlying mechanism; VT is frequently associated with myocarditis, congenital heart diseases, complete heart block, and congenital long QT syndrome. In our case, long QT syndrome was not confirmed, and there were no data to support myocardial inflammation at the time of fetal VT. In addition, a negative history for intrauterine infection, rapid control of VT after starting oral treatment with sotalol, with significant reduction in the effusion and ascites, and immediate postnatal ECG and echo exam were normal. The inflammatory syndrome became evident on the third day from the time of diagnosis of SARS-CoV infection, being associated with coagulopathy and the positiviation of the myocardial enzymes. In the second week of COVID infection, echocardiographic evaluation showed impaired LV function and a tendency towards dilatation of the coronary arteries, correlated with the maximum serum levels of the cardiac enzymes (TnI and CK-MB). We consider that, in our case, there is a significant overlap between fetal VT, associated with impaired longitudinal strain values diagnosed at 33 weeks of gestation, and COVID infection, diagnosed after birth; both factors contribute to myocardial dysfunction with fulminant clinical evolution. As a consequence, the clinical impact of the single factors is not easily assessable. However, the myocardial inflammatory process associated with SARS-CoV infection contributed to the postnatal arrhythmic storm. Therapeutic control of VT, along with improvement in inflammatory, cardiac biomarkers and cardiac function, in response to therapy with immunoglobulin, glucocorticoids and levosimendan, support this hypothesis. Anti-inflammatory therapy and immunoglobulin therapy were important in the management of this case. Zhu H et al. showed that early administration of immunoglobulin may reduce neonatal morbidity and mortality [17]. The presence of intense systemic inflammation in acute SARS-CoV myocarditis has been proven [2,5]. The use of immunosuppressive therapy with glucocorticoids has been shown to improve cardiac function in myocarditis and inflammatory cardiomyopathy [18]. In our case, the markers of myocardial injury dropped significantly after 3 weeks of treatment, with significant improvement in LV parameters.

Several studies underline the importance of IL-6 level quantification in COVID patients, as it can assess disease severity and predict the outcome [19,20]. Due to limited financial resources, the determination of IL-6 levels could not be performed in our case. Another limitation in the diagnostic process was imposed by the impossibility to perform a cardiac MRI at our center in the neonatal period, although the imaging technique is currently considered to be the non-invasive gold standard in the diagnostic protocol of myocarditis, through the detection of myocardial injury [21]. What makes our case different from the newborns reported by other authors are the following aspects: the clinical picture with VT, the rapid progression to coronary injuries with progressive dilation of the left coronary artery, the myocardial dyskinesia and decreased LV ejection fraction on echocardiography, and the absence of consistent respiratory symptoms. To our knowledge, this is the first case describing neonatal myocardial dysfunction associated with SARS-CoV infection complicating the clinical course of rare fetal tachyarrhythmia.

## 4. Conclusions

In view of the current epidemiologic situation, SARS-CoV should be considered as a possible cause in other clinical conditions, such as myocardial inflammation, even in the absence of respiratory symptoms. Our case highlights the potential of myocardial involvement in a severe clinical course of COVID infection, mostly in infants with comorbidities.

Close follow-up and repeated echocardiographic examinations are essential in detecting cardiac complications such as myocardial dysfunction and coronary artery dilation.

## Figures and Tables

**Table 1:** Laboratory findings.

Test	Reference Range	Day 3 after Birth	Day of Illness										
			3	5	7	10	11	12	13	15	19	24	27
White blood cell	5–20 × 10³/µL	8.85	13.03	13.67	-	14.44	-	-	9.83	11.45	10.33	12.37	10.50
Lymphocytes	2–17 × 10³/µL	3.44	3.23	7.48	-	4.64	-	-	3.05	4.13	3.33	2.06	2.84
Eosinophils	0.03–0.4 × 10³/µL	0.41	0.00	0.42	-	0.2	-	-	0.33	0.34	0.01	0.01	0.03
Basophils	0.01–0.08 × 10³/µL	0.03	0.01	0.01	-	0.06	-	-	0.09	0.06	0.02	0.01	0.01
Hemoglobin	15.5–21.5 g/dL	17.00	14.1	14.60	-	13.40	-	-	11.3	11.8	11.1	9.9	11.3
Hematocrit	44–60%	47.80	41.1	42.10	-	38.10	-	-	33.9	34	32.1	29.1	30.5
Platelets	150–450 × 10³/µL	246	391	329	-	659	-	-	812	942	939	829	517
CRP	0–5 mg/L	1.34	26.00	9.86	10.11	3.58	-	-	0.80	0.38	0.37	-	0.30
Procalcitonin	<0.5 ng/mL	-	0.28	-	-	-	-	-	-	-	-	negative	-
Ferritin	15–120 ng/mL	-	-	-	339.00	351.40	-	443.80	-	401.90	370.00	-	-
LDH	125–220 U/L	-	-	-	-	-	411	-	-	195	-	257.00	297
D-dimer	<400 ng/mL	762.00	729	1402.00	2559.60	949.66	-	-	1005.53	496.59	-	<200	-
Tn-I	0–15.6 pg/mL	7.5	25.70	50.90	117.40	87.50	-	-	134.70	48.30	24.40	-	negative
CK-MB	0–24 U/L	42.70	16.70	44.20	74.00	37.10	-	42.90	-	31.70	25.60	-	38
CK	29–168 U/L	284.00	48.00	56.00	-	31.00	-	38.00	-	29	29.00	20.00	31
NTproBNP	<450 pg/mL	-	-	-	-	-	-	645.40	-	-	140.22	-	22.91
AST	0–33 U/L	56.00	19.00	-	-	-	35.00	33.00	-	-	-	22.00	24
ALT	0–55 U/L	38.00	17.00	-	-	-	39.00	41.00	-	29	34.00	26.5	32
Urea	10.91–40.66 mg/dL	7.70	7.50	11.80	-	-	36.00	48.80	40.70	47.1	55.00	57.5	47
Creatinine	0.57–1.11 mg/dL	0.55	0.70	0.59	-	-	0.50	0.63	0.59	0.61	0.52	0.43	0.48
Albumin	3.8–5.4 g/dL	-	-	3.10	-	-	-	-	-	-	-	-	3.9
Lactic acid	0.9–1.7 mmol/L	2.1	-	1.7	-	2.6	1.2	2.5	1.9	1.9	-	-	3.95
Na	133–146 mmol/L	139.00	159.00	140.00	-	-	133.00	-	125	131	131.00	136.00	128.8
K	3.7–5.9 mmol/L	3.63	4.80	6.36	-	-	5.60	-	5.75	4.68	6.49	-	3.61

CRP: C-reactive protein; LDH: lactate dehydrogenase; Tn-I: troponine I; CK-MB: creatine kinase-MB (determined by kinetic method); CK: creatine kinase; NTproBNP: N-terminal fraction of pro-brain natriuretic peptide; AST: aspartate aminotransferase; ALT: alanine aminotransferase; Na: natrium, sodiu; K: potassium.

## Data Availability

The raw data presented in this study can be obtained upon reasonable request addressed to Liliana Gozar (lili_gozar@yahoo.com).
